# Multivariate Analysis of Fecal Metabolites from Children with Autism Spectrum Disorder and Gastrointestinal Symptoms before and after Microbiota Transfer Therapy

**DOI:** 10.3390/jpm10040152

**Published:** 2020-10-02

**Authors:** Fatir Qureshi, James Adams, Kathryn Hanagan, Dae-Wook Kang, Rosa Krajmalnik-Brown, Juergen Hahn

**Affiliations:** 1 Biomedical Engineering, Rensselaer Polytechnic Institute, Troy, NY 12180, USA; quresf2@rpi.edu; 2 Center for Biotechnology and Interdisciplinary Studies, Rensselaer Polytechnic Institute, Troy, NY 12180, USA; 3 School for Engineering of Matter, Transport, and Energy, Arizona State University, Tempe, AZ 85287, USA; Jim.Adams@asu.edu; 4 Department of Computer Science, Purdue University, West Lafayette, IN 47907, USA; khanagan@purdue.edu; 5 Biodesign Swette Center for Environmental Biotechnology, Arizona State University, Tempe, AZ 85287, USA; DaeWook.Kang@utoledo.edu (D.-W.K.); Dr.Rosy@asu.edu (R.K.-B.); 6 Biodesign Center for Health through Microbiome, Arizona State University, Tempe, AZ 85287, USA; 7 School of Sustainable Engineering and the Built Environment, Arizona State University, Tempe, AZ 85281, USA; 8 Department of Chemical and Biological Engineering, Rensselaer Polytechnic Institute, Troy, NY 12180, USA

**Keywords:** fecal metabolites, ASD, microbiome, gastrointestinal symptoms, Fisher Discriminant Analysis

## Abstract

Fecal microbiota transplant (FMT) holds significant promise for patients with Autism Spectrum Disorder (ASD) and gastrointestinal (GI) symptoms. Prior work has demonstrated that plasma metabolite profiles of children with ASD become more similar to those of their typically developing (TD) peers following this treatment. This work measures the concentration of 669 biochemical compounds in feces of a cohort of 18 ASD and 20 TD children using ultrahigh performance liquid chromatography-tandem mass spectroscopy. Subsequent measurements were taken from the ASD cohort over the course of 10-week Microbiota Transfer Therapy (MTT) and 8 weeks after completion of this treatment. Univariate and multivariate statistical analysis techniques were used to characterize differences in metabolites before, during, and after treatment. Using Fisher Discriminant Analysis (FDA), it was possible to attain multivariate metabolite models capable of achieving a sensitivity of 94% and a specificity of 95% after cross-validation. Observations made following MTT indicate that the fecal metabolite profiles become more like those of the TD cohort. There was an 82–88% decrease in the median difference of the ASD and TD group for the panel metabolites, and among the top fifty most discriminating individual metabolites, 96% report more comparable values following treatment. Thus, these findings are similar, although less pronounced, as those determined using plasma metabolites.

## 1. Introduction

Autism spectrum disorder (ASD) encompasses a large group of early onset neurological conditions that result in impairments in social behavior and communication, which are estimated to affect 1 in 54 children under the age of eight in the United States [1]. Despite this high rate of occurrence, the understanding of the pathophysiology of ASD is still poor, and it is believed that at least in some cases ASD begins prenatally as a result of complex interactions between environmental and genetic factors [2,3]. Although diagnosis of this disorder is only made through behavioral evaluations, many systems of the body are strongly affected by this condition. A diverse range of physiological mechanisms have been observed to be perturbed in ASD including the immune, endocrine, and gastrointestinal (GI) systems [4,5]. Notably, the prevalence of GI symptoms co-occurring with ASD (~46%) lends significant credence to investigating the relationship of ASD to the GI system [6].

In recent years, there have been growing efforts to study the effect of the microbiome on the Gut-Brain Axis in the context of ASD etiology. Some studies have shown that the gut microbiome of individuals with GI issues varies significantly from those without such complications [7,8,9]. However, the microbiota of individuals with ASD without the presence of GI issues have also consistently been found to be distinct from their typically developing (TD) peers [10,11]. Certain genera such as Prevotella and Coprococcus have been shown to be significantly less prevalent in the gut of children with ASD [12,13]. Furthermore, it has been proposed that the microbiota differences in children with ASD give rise to metabolomic differences that can be quantitatively evaluated to distinguish them from their TD peers [14,15].

Some previous work involving fecal metabolites identified isopropanol, p-cresol, acetyl-carnitine, free carnitine and neurotransmitters-gamma-Aminobutyrate (GABA) as metabolites that have significantly different concentrations between the ASD and TD cohorts [14,16,17]. There have also been mixed results regarding the fecal concentrations of short chain fatty acids. While some studies show that the fecal concentration of acetic, propionic and butyric acids were higher in children with ASD [18,19,20], other investigations found that the concentration of these short chain fatty acids were lower or comparable to their TD peers [21,22,23].

As the role of the microbiome in ASD is being in more detail, the question is raised as to whether using fecal microbiota transplant (FMT) can mitigate the severity of GI and other symptoms of ASD. In one notable study, offspring of germ-free mice subject to microbiome transfer from individuals with ASD exhibited more ASD-like behaviors and produced different metabolome profiles when compared to offspring of germ-free mice subject to microbiome transfer from TD controls [24]. The use of FMT has shown considerable potential in its capability to alleviate not only symptoms associated with GI complications, but also in some cases to reduce the severity of certain behavioral symptoms in children with ASD. For example, Kang et al. demonstrated in an open-label study that through a modified FMT (called, Microbiota Transfer Therapy (MTT)), there was an 80% reduction in GI symptoms and a 24% initial reduction in core ASD symptoms, with greater improvement in ASD symptoms at a two-year follow-up [25,26]. Probiotic intervention has also shown potential to have a positive influence on ameliorating both behavioral and GI symptoms in individuals with ASD [27,28].

Past work in analyzing metabolites prior and subsequent to MTT therapy have also yielded promising results. Children with ASD who underwent MTT presented changes in their plasma metabolite profiles to resemble more closely those of their typically developing peers [29,30]. The work presented in this paper builds on the analysis of this same study [25], but focuses on fecal metabolites instead of plasma metabolites. Univariate assessment of the fecal metabolites examined in this study have previously shown limited capability for differentiating between ASD and TD cohorts when corrected for multiple hypotheses [30]. Thus, here we explored the use of multivariate techniques to detect underlying relationships that may have been otherwise missed.

## 2. Materials and Methods

### 2.1. Study Design

The purpose of this study was to examine the differences in gut metabolites between children with ASD and GI problems vs. typically developing children without GI problems, and determine the effects of gut microbiota transfer therapy on the fecal metabolites of the ASD group. The study involved 38 children, aged 7–16 years, 18 of these professionally diagnosed with ASD by a healthcare provider (verified with the Autism Diagnostic Interview-Revised) and 20 determined to be typically developing. The participants with ASD were required to have moderate to severe GI problems, and the range of GI issues included constipation, diarrhea, and alternating diarrhea/constipation. GI symptoms were assessed biweekly with the Gastrointestinal Severity Rating Scale (GSRS) and daily with a Daily Stool Record using the Bristol Stool Form scale [25]. The study consisted of 2 weeks of antibiotic therapy, 1 day of bowel cleans, and a high major initial dose and 7–8 weeks of lower maintenance doses of FMT treatment followed by evaluation at 8 weeks post treatment. The TD group did not undergo MTT, but instead was used as a comparison group whose measurements were taken at the same time as the ASD group before treatment. The MTT experimental protocol and details of the study population are outlined in Kang et al. [25].

The pre-treatment protocol consisted of two weeks of oral vancomycin, which is a broad spectrum non-absorbable antibiotic. This treatment was intended to reduce pathogenic bacteria and prime the GI system for MTT. The dose of vancomycin administered was individualized to the weight of each participant at 40 mg/kg, with a maximum dose of 2 g [25]. Participants were then subjected to one day of fasting and a bowel cleanser (MoviPrep) in order to remove the vancomycin and further reduce levels of intestinal bacteria. Standardized Human Gut Microbiota (SHGM) consisted of a full spectrum of highly purified microbiota from healthy, carefully screened donors. The ASD cohort was split into two groups, each one following a different initial high dose (2.5 × 10^12^ cells/day) SHGM treatment. One MTT treatment consisted of a single dose administered rectally (*n* = 6) while the other involved doses administered orally on two days (*n* = 12). Both techniques were followed by a lower concentration SHGM maintenance dosage (around 2.5 × 10^9^ cells) given orally, with treatment ending 8 weeks after the initial high dose [25]. However, the protocol differed slightly for both groups of ASD children as those that received SHGM rectally waited for one week prior to beginning low dose SHGM.

### 2.2. Metabolite Measurements

Once the study had concluded, aliquots of the fecal samples were shipped overnight on dry ice to Metabolon (Durham, NC, USA). Both the control and autism samples were blinded and randomized prior to being shipped. Metabolon utilized ultrahigh performance liquid chromatography-tandem mass spectroscopy (UHPLC-MS/MS) instruments for obtaining metabolomic information on 669 metabolites. A detailed overview regarding this protocol can be found in Long et al. [31]. By using this technique, it is possible to determine a signal intensity corresponding to a metabolite’s presence in a sample. Subsequently, the signal intensity is used to derive the relative abundance of each metabolite. For this objective, peak area integration using the area under the curve was utilized. In the case of missing values, imputation was performed by taking the lowest value of each compound measurement divided by the square root of 2.

**Figure 1:**
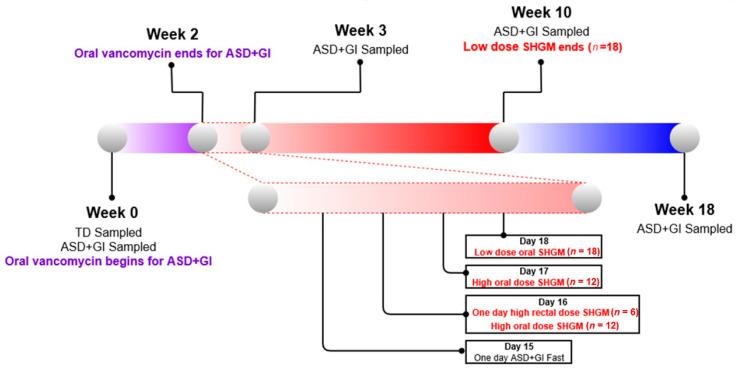
Timeline for the experimental protocol, which can be divided into three main phases. From Week 0 to Week 3, the Autism Spectrum Disorder (ASD) cohort is primed for Microbiota Transfer Therapy (MTT). From Week 3 to Week 10 the ASD cohort receives low dose fecal microbiota (FM) or is prepared for low dose FM, and finally from Week 10 to Week 18 no treatment is given to the individuals. A closeup is provided of Week 2–3 as this is when Standardized Human Gut Microbiota (SHGM) is initialized.

Fecal samples were taken at four time points from the participants with ASD (Figure 1). Parents were instructed to freeze these sample immediately after collection for up to 3 days, and the samples were then transported to Arizona State University on dry ice where they were stored in a −80 °C freezer. Initial fecal samples were collected from all participants at Week 0. Samples were also taken from ASD participants at the Week 3 mark from the beginning of the treatment (after about five days of microbiota transplant) and at the end of MTT treatment (Week 10). The ASD group was sampled again 8 weeks after administration of SHGM ceased (Week 18). In total, 18 ASD participants collected samples at all time points aside from Week 3, where only 17 samples were collected. The TD group received no treatment and 20 were sampled at the beginning (Week 0).

### 2.3. Statistical Analysis

The data collected for each of the metabolites underwent various forms of statistical analysis to assess differences between the ASD + GI and TD cohort. By comparing the differences observed for metabolites before and after MTT, it might be possible to gain some understanding of the role that this therapy could play in altering metabolic processes. Both univariate and multivariate techniques were used in this regard, and the implementation of the analysis routines was done in MATLAB.

#### 2.3.1. Preprocessing

In order to ensure continuous distribution of values across all participants, metabolites with too many values below the detection limit at their initial stool sample (Week 0) were removed. The detection limit for a metabolite was determined to be the minimum value recorded for that metabolite. If less than 40% of all measurements were above the detection limit, the metabolite was removed from subsequent analysis. This step accounted for the possibility that a measurement could be almost entirely below the detection limit in one cohort, while simultaneously being above the limit in the other cohort. The remaining metabolites were then normalized such that for each metabolite the median value was 1.0 in the Week 0 TD cohort.

#### 2.3.2. Univariate Analysis

Univariate analysis identifies metabolites that are differentially expressed between the ASD and TD cohorts. Using this information, it is possible to examine common correlations and relationships across different measurement quantities. In turn, this has the potential to identify underlying mechanisms of ASD etiology as well as provide guidance for the development of a multivariate model that can more accurately distinguish between both groups. As there are 669 metabolites under investigation, there is significant concern related to overfitting of statistical models if many or all these measurements are used to develop a multivariate model. By reducing the number of measurements to a smaller subset, it is possible to alleviate some of the concerns related to overfitting.

Metabolites were individually analyzed for their ability to classify between the ASD and TD cohorts at their Week 0 measurements. The area under the receiver operator curve (AUROC) served as an assessment of the potential of a metabolite to distinguish between ASD and TD groups. This metric is defined as the false positive rate against the false negative rate at different ASD/TD classification thresholds. An AUROC of 1.0 indicates the capacity for perfect separation, while an AUROC of 0.5 indicates that there is no ability to distinguish between the groups. Metabolites with an AUROC value above 0.6 were selected as candidates for use in multivariate analysis in this work.

Univariate analysis techniques evaluated whether significant changes had occurred among the metabolites between the beginning and end of the study for the ASD cohort. For this purpose, the metabolite measurements at Week 0 and Week 18 were compared using a parametric or non-parametric test, depending upon their distribution. An Anderson-Darling test for normality was used at both time points to determine the distribution of each set of measurements. Subsequently, either a Wilcoxon signed-rank test or a paired t-test was performed on the ASD group, comparing measurements from Week 0 to Week 18. A relatively normal distribution employs the parametric paired t-test; otherwise, the non-parametric Wilcoxon signed-rank test is used. The resulting *p*-value indicates how significantly the concentration of the metabolite changed for the cohort over the course of the study.

As there were a considerable number of quantities measured per study participant, it was imperative that multiple hypothesis correction tests were utilized. Subsequently, a false discovery rate (FDR) for each individual metabolite was computed using leave-n-out (*n* = 1, 2, 3) cross validation (see Table A1). Leave-n-out is an iterative process and involves removing n individual data points from the total dataset and rerunning the univariate analysis on this subset. This procedure is repeated so that all possible combinations with n removed individuals are assessed. The FDR is calculated as the proportion of univariate results that were not deemed significant.

#### 2.3.3. Multivariate Analysis

Fisher discriminant analysis (FDA), metabolites that had been identified as having an AUROC value above 0.6 were used to develop a multivariate model for distinguishing between the ASD and TD cohorts. FDA is a dimensionality reduction technique that seeks to separate classes of data by determining a projection where such separation is maximized [32]. This is achieved by maximizing the ratio between the between-class variability *S_B_* and the within-class variability *S_W_* for a weight vector ***W***.

$$J\left(W\right)=\frac{W^{T}S_{B}W}{W^{T}\;S_{W}\;W}$$

In the case of K classes with n number of samples and m measurements, *S_B_* is defined as follows, where $\bar{x}$ denotes the global mean, ${\bar{x}}_{k}$ denotes the local class mean, and *n_k_* is the number of samples within class *k*:

$$S_{B}=\overset{K}{\underset{k=1}{\sum }}n_{k}\left({\bar{x}}_{k}-\bar{x}\right){\left({\bar{x}}_{k}-\bar{x}\right)}^{T}$$

In contrast, the within-class covariance matrix *S_W_* is defined as the following, where *x_i_* corresponds to an individual sample:

$$S_{W}=\overset{K}{\underset{k=1}{\sum }}n_{k}\underset{i\in k}{\sum }\left({\bar{x}}_{i}-{\bar{x}}_{k}\right){\left({\bar{x}}_{i}-{\bar{x}}_{k}\right)}^{T}$$

Thus, FDA simultaneously maximizes the scatter between classes and minimizes the scatter within each class to find *k*-1 vectors that maximize the objective function. Subsequently, the eigenvector corresponding to the *k*-1 largest eigenvalue of $S_{B}$*S_W_* corresponds to the optimum weight vector.

For this dataset, the objective of FDA is to separate the ASD and TD cohorts with a combination of metabolites. The initial stool samples (Week 0) were used to develop these models, so that the model classifies individuals before any treatment.

The previously mentioned preprocessing and univariate analysis steps were performed to reduce the set of metabolites considered for FDA. An FDA model could potentially be created with all 669 metabolites, but this model would likely overfit the data. To account for this, only metabolites that passed the preprocessing step and achieved a univariate AUROC of over 0.6 remained in consideration. This resulted in 165 metabolites under further investigation.

An exhaustive search was performed through all possible combinations of 2, 3, or 4 metabolites of the reduced set of 165 metabolites to determine the models which best separate the ASD and TD groups at Week 0. The AUROC was used here as well, measuring how well the multivariate models classify the two groups. Using kernel density estimation, the probability density function of each model was computed. Iterating through all combinations, the models were assessed, and the combination of metabolites was determined for each number of variables. For each number of metabolites, the 1000 models that had achieved the highest possible AUROC were recorded. To derive the five-metabolite models, all 1000 four metabolite models that had achieved the highest AUROC were augmented with each of the remaining 161 metabolites that had an AUROC greater than 0.6. The top 1000 five metabolite models that had the highest AUROC were then subjected to leave-one-out cross validation.

### 2.4. Cross-Validation

Leave-one-out cross-validation was performed on the optimal FDA models to evaluate robustness and statistical independence. Cross-validation ensures that, rather than merely fitting a model to presented data, the model obtained is also capable of classifying new data. Although cross-validation generally has a lower accuracy than what is computed just by fitting a model to data, the cross-validation accuracy will better reflect generalizability to new data sets, i.e., data not used for developing a classifier. Leave-one-out cross-validation proceeds iteratively, as a single individual’s data is removed from the total dataset, then an FDA model is computed with measurements from the remaining individuals [33]. The measurements from the removed individual are now used as a test case to determine if the model prediction regarding classification is correct. This process is repeated for measurements from each of the individuals in the dataset: their data are removed, a model is developed with the remaining data, then they are classified with this model, until the data for each individual has been removed once. A confusion matrix is computed which includes the true positive rate (TPR), or sensitivity, and the true negative rate (TNR), or specificity. Additionally, for each model, the Type II (false negative) error β was modulated between 0.01, 0.05, 0.1, and 0.2 during cross-validation. The Type II error determined the threshold value for separating the two groups. By alternating the values of β, it was possible to evaluate the cross-validated performances along different positions of the ASD distribution. Lowering β meant raising the Type I error while lowering the Type II error and the converse also holds true. Thus, each of the four models (2, 3, 4, or 5 metabolites) had cross-validation performed four times, with corresponding computation of TPRs and TNRs.

### 2.5. Model Evaluation

The models obtained after cross validating at different thresholds for data collected at Week 0 were used to make predictions about the ASD group at the other MTT time points. Specifically, measurements at Week 3, Week 10 and Week 18 were used to monitor the change in classification performance over the course of the MTT protocol. Data from these time points were rescaled with respect to the TD Week 0 median and standard deviation. The probability density functions were compared between the time points, and the discriminate scores for each model as well as of their constituent metabolites were determined. Changes resulting from MTT were quantified using the Type II error, with respect to the threshold associated with the probability density function (PDF) of the ASD + GI cohort’s discriminant scores at each time point. Thus, both univariate assessments were performed as well as the total assessment of the multivariate models’ discriminant score. Additionally, correlation analysis between significant metabolite pairs was performed to determine possible underlying relationships.

## 3. Results

### 3.1. Univariate Analysis

In total, there were 669 fecal metabolites that were measured in the study. Through the preprocessing step, 86 metabolites were determined not to have the prerequisite number of observations above the detection limit for further analysis. In order to classify ASD and TD cohorts at their Week 0 measurements, the area under the AUROC was used as an assessment of the potential of a metabolite to distinguish between ASD and TD groups. The remaining 583 metabolites were ranked according to their univariate AUROC, and 165 metabolites with an AUROC of at least 0.6 were identified. No single metabolite perfectly separated the cohorts (which would correspond to an AUROC = 1.0), as the metabolite with the highest AUROC, carnitine, achieved a value of 0.77 (Table 1).

Using the 165 metabolites with an AUROC greater than 0.6, additional univariate testing was performed to assess the degree to which measurements shifted following MTT. ASD metabolite samples measured at Week 0 were compared to their values following MTT at Week 18 using either a paired t-test or a Wilcoxon signed-rank test depending upon the distribution determined for the data via the Anderson-Darling normality test. It was found that 10.9% of the metabolites significantly changed (*p* < 0.05) following the MTT therapy when comparing the ASD group before and after treatment (see Table A1). The metabolites that had a threshold AUROC value of 0.6 were subsequently used for model discovery for the 2-, 3-, 4- and 5- metabolite models.

### 3.2. FDA Models

**Figure 2:**
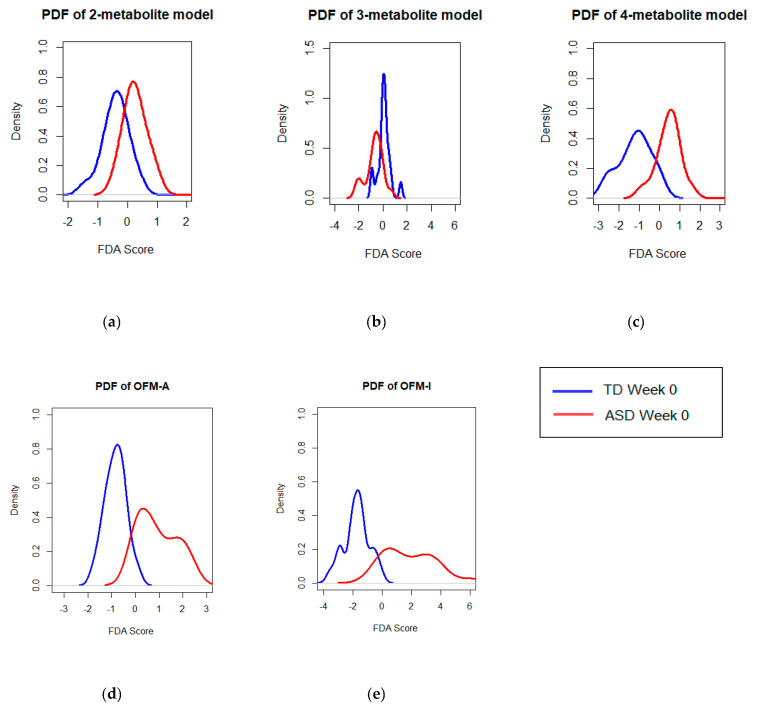
PDFs of ASD and TD discriminant scores at Week 0. The probability density function of the FDA score provides a visualization of a model’s ability to distinguish between the ASD and TD cohorts. The (**a**) two-metabolite model has most of its FDA scores highly concentrated near the region where thresholds would be applied. The (**b**) three-metabolite model is not as highly concentrated, but there is a significant amount of overlap between the scores of the ASD and TD participants, which is visible in both plots. The four (**c**) and five (**d**,**e**) metabolite models better separated the cohorts, with little overlap in the discriminant scores of the ASD and TD groups.

The FDA models with the greatest AUROC values for each number of constituent metabolites are listed in Table 2. The probability density function (PDF) of discriminant scores for the 2-, 3-, 4-, and 5-metabolite models that achieved the highest accuracy following cross validation are shown in Figure 2. There were two distinct models that were identified, using five separate metabolites, as having achieved the same accuracy after cross-validation. With the exception of one metabolite which differed between them (Adenosine and Indole), the constituents of these panels are identical. These two metabolite models are both shown in Table 2 and will be referred to as OFM-A and OFM-I, optimized fecal model-adenosine and optimized fecal model-indole, respectively (OFM-I/A). For all optimized metabolite panels, the TPR and TNR values for each are presented when the β value was modulated. The 5-metabolite models had higher AUROCs than the 2-, 3-, and 4-metabolite models, so they are the focus of the following analysis, due to their higher accuracy (0.95 specificity and 0.94 sensitivity). Modulating β revealed that the optimal cut-offs between the ASD and TD distributions for the OFM-I and OFM-A models was β = 0.05 for both the OFM-A and OFM-I.

**Figure 3:**
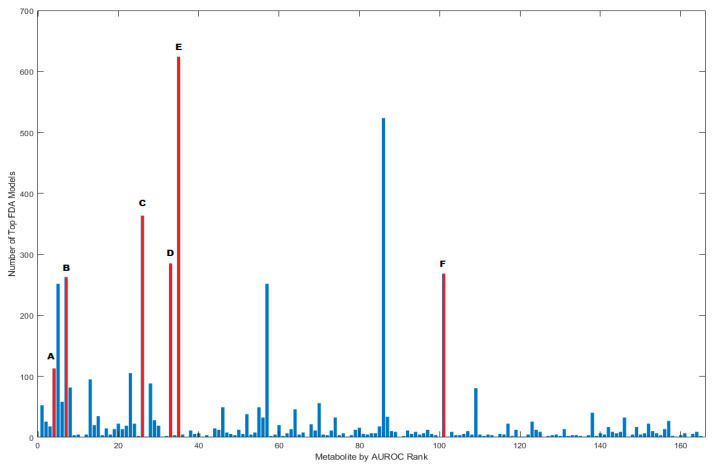
Frequency of appearance of each of the metabolites with AUROC > 0.6 in the top 1000 five-metabolite Fisher discriminant analysis (FDA) models. The metabolites are ranked from highest to lowest area under the receiver operating characteristic curve (AUROC) as shown in Table 1. The metabolites included in the FDA models which achieved maximal separation following cross-validation are shown in red: (A) indole (B) imidazole Propionate (C) adenosine (D) theobromine (E) hydroxyproline, (F) 2-hydroxy-3-methylvalerate.

For the 1000 best models with five metabolites, the AUROC ranged from 0.97 to 1.00 which are high values. The reason for using the 1000 best models is that there are not only one or two best models as judged by AUROC alone. Each of these models was subjected to cross validation, with OFM-I/A being derived from those that achieved the highest accuracy. The metabolites ultimately utilized for the development of a five-component model were all found to be in the top quartile of prevalence in the 1000 top models (Figure 3). Notably, among the top fecal metabolite models, adenosine and hydroxyproline appeared in 36.3% and 62.4% of models, respectively. Only three metabolites were present in more than 25% of the top 1000 models that were not among those included in the OFM-I/A panels. These metabolites were Adenine, 2-aminobutyrate and 1,7-dimethylurate (corresponding to the 5th, 57th, and 86th highest AUROC rank, respectively).

### 3.3. Correlation Analysis

Correlation analysis was performed on the OFM metabolites as these were the ones that had been identified as being able to distinguish between the ASD and TD cohorts with the highest accuracy after cross-validation. It can be observed that many of the top 50 metabolites (AUROC ≥ 0.66) were significantly correlated with the OFM metabolites (Table 3). In contrast, the individual OFM metabolites for both models had little to no correlation with each other, apart from hydroxyproline with adenosine and 2-hydroxy-3-methylvalerate; these findings were expected since, if individual OFM metabolites were highly correlated with each other, then they would not be useful in the model due to their correlation.

### 3.4. Assessing Effects of MTT

Univariate assessment of the top 50 metabolites as ranked by AUROC demonstrated that 14% of these 50 metabolites showed significant differences in their Week 0 and Week 18 ASD measurements and that 47 of these 50 metabolites achieved a lower AUROC eight weeks following treatment (Table 1). In addition to classification at baseline, the multivariate models developed can be used to observe changes in fecal metabolome composition over the course of the study. Most metabolites in the OFM-I and OFM-A models changed significantly after MTT and have values closer to the TD group after MTT (see Table 4). The average difference between the median of the five metabolites for TD group at Week 0 and the ASD measurements at Week 18 compared to measurements at Week 0 diminished by 88% and 82% for the OFM-I and OFM-A models (see Table 4), so the ASD group became much more similar to the TD group.

**Figure 4:**
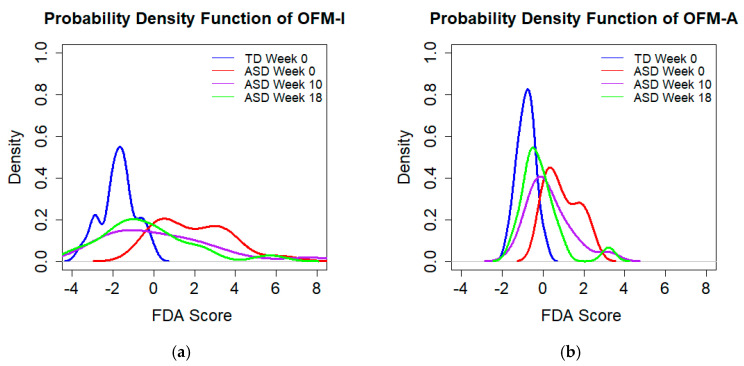
PDF curves for the (**a**) OFM-I and (**b**) OFM-A model when assessing the ASD cohort over the course of the study. The overlap between the TD cohort and the ASD cohorts increases at Week 10 and Week 18, indicating that the metabolite profile of the ASD group after MTT treatment has become more similar to the ones of the TD group.

The OFM-I/A models were applied to the ASD samples at all distinct time points to assess their accuracy for classifying a sample as belonging to the ASD or TD cohort. The effectiveness of OFM-I/A for classification changed significantly before and after MTT. The type II error rate was initially observed to be 5% for both models, indicating that the ASD and TD distributions are quite distinct, but was observed to rise to 56% eight weeks after MTT was completed (Table 4), thereby indicating that distinguishing between the ASD and TD cohort is not reliably possible after MTT. The PDF curves are shown in Figure 4 to demonstrate the changes in the ASD cohort over time with respect to the values of the FDA score. The distributions indicate that the ASD cohort became more metabolically similar to the TD cohort after treatment, since the curves are shifted towards the TD curve. Notably, the distribution of scores for the ASD cohort become somewhat bimodal at the later time points for both models. The discriminant score for both models decreased substantially as time progressed, indicating that the metabolites of the ASD group were becoming more similar to that of the TD group.

## 4. Discussion

Preliminary analysis using univariate methods revealed that none of the individual fecal metabolites achieved a high AUROC value by itself. Interpretations regarding the threshold value needed for an AUROC to be deemed an effective classifier vary by discipline. AUROC values between 0.9–1.0 are desirable for diagnostic tests and are seen to be reflective of excellent classification [35]. However, this value was not chosen here as the AUROC is employed here as a pre-screening tool for reducing the number of metabolites for classification, and not for determining a metabolite that by itself can distinguish between the two groups. The highest AUROC value for an individual metabolite was 0.77, corresponding to carnitine, indicating that the ASD group is somewhat heterogeneous. In contrast, all optimized multivariate models using three or more elements were able to achieve an AUROC greater than 0.9, highlighting that a multivariate analysis can provide better classification than that which can be determined using univariate analysis alone. Nonetheless, 94% of the top 50 univariate metabolites report lower AUROC (Table 1) eight weeks following MTT, which indicates greater similarity between the ASD and TD measurements after treatment.

Analysis of all possible significant metabolites at Week 0 resulted in the OFM-I and OMF-A models, consisting of five metabolites. Four of these five metabolites were identical between the two models and both achieved AUROC values greater than 0.99. Interestingly, the two metabolites that differed between them, Adenosine and Indole, are associated with different metabolic processes and have no significant correlation. Furthermore, cross-validation revealed that using the OFM-I/A models at the Week 0 timepoint resulted in a 0.95 TPR and 0.94 TNR. Subsequently, there was an overall 94.7% accuracy for correctly classifying an individual into the ASD/TD groups after leave-one-out cross-validation.

Many of the metabolites identified as being differentially expressed between the ASD and TD cohorts have also been previously examined for their relationship to ASD. Specifically, among the top five metabolites ranked by their AUROC value, carnitine, indole and sphingosine have all been found to be differentially expressed in some capacity among individuals with ASD [14,17,18,36]. In a meta-analysis, 10–20% of individuals with ASD were found to have disorders with synthesizing carnitine, which was the metabolite that had achieved the largest AUROC value [36]. Plasma carnitine concentration has also previously been shown to be lower among cohorts with ASD [36]. It should be noted that following MTT the AUROC values of carnitine reduced to 0.68, which indicates that the ASD and TD carnitine distributions were less different after MTT (see Table A1). Sphingolipids such as sphingosine have been found to play an active role in the crosstalk between microbiota and intestinal cells [37]. The significant change in concentration for metabolites such as N-palmitoyl-sphingosine (d18:1/16:0) may have been associated with the changed microbiome composition resulting from MTT (Table 1). Approximately 68% of the variance observed in the fecal metabolome can be explained by the gut microbiome [38], which underscores the potential impact FMT can have on reshaping metabolite concentrations.

Among the metabolites which form part of the OFM-I/A models, theobromine exhibited a significant change between its measurements at Week 0 compared to the Week 18 value for the ASD cohort when a sign ranked test was applied at both timepoints (Table 1). Theobromine is not a microbial metabolite, and its source in fecal samples likely stems from dietary intake and from human metabolism of caffeine [38]. Consequently, this may account for the reason why it was not observed to be correlated with any other metabolite and why the median discriminate score often took the value of the detection limit. However, the metabolization of theobromine is primarily via hepatic demethylation and oxidation, which are processes that have at least been hypothesized to be perturbed in ASD [39,40]. The median concentration for this metabolite was also not found to change following the bowel cleanse measurement (see Table A1). We conducted a secondary analysis of a five-metabolite model without theobromine and found that it results in significantly lower sensitivity and specificity, so including theobromine seems to be important for developing a classification model.

Nonetheless, in the case of all metabolites present in the OFM-I/A models, the average difference between the Week 0 TD measurements and ASD group decreased greatly (82–88%) by the end of the study (Table 4). Hydroxyproline, which is another of the OFM metabolites, has been previously determined to be expressed in significantly higher concentration in the plasma of children with ASD, consistent with the higher levels in feces [41] and in the present study. Indole, which was also one of the OFM metabolites, has been found in higher concentration in fecal samples in children with ASD and other neurodevelopmental conditions [17], consistent with the results of this study, and is an important metabolite for tryptophan metabolism [42]. Thus, the shift to a lower discriminant score following the completion of the treatment is consistent with measurements of ASD fecal metabolites becoming more like those of their TD counterparts following MTT.

The OFM-I/A models in their totality demonstrated similar behavior when contrasting measurements taken at Week 0 and Week 18 of the ASD cohort. This study found that some metabolic changes had begun by Week 3 (after vancomycin, bowel cleanse, and approximately five days of FMT). It is also notable that the distributions of FDA scores within both the five-metabolite models at later timepoints (Week 10 and Week 18) are bimodal. This suggests that some individuals may respond differently to MTT than others. This finding was similar to the analysis performed on plasma metabolites where a steep decline in median discriminant score was also observed at Week 3 and Week 10 [29].

The OFM-I/A metabolites demonstrated limited correlation among themselves. This was to be expected as FDA seeks to maximize the amount of discriminating information with a minimal number of utilized metabolites. For this reason, within this subset of fecal metabolites, those with few correlations tended to appear more frequently in the top 1000 models. Specifically, there was a high proportion of top 1000 models featuring theobromine (28.5%), which was ranked as the fourth most common metabolite present in the models. Notably, this metabolite was not significantly correlated with any of the other top 50 metabolites as discussed above. In total, 44 of the 50 metabolites with the highest AUROC were correlated with the OFM metabolite panel, suggesting that there are at least six common types of metabolic abnormalities associated with ASD. Although adenosine and indole were not found to be correlated, they were included in the OFM-A and OFI-I models, respectively, and both metabolites are related to distinct biological pathways, with adenosine being associated with purine metabolism, while indole is associated with tryptophan metabolism. It was observed in one study that metabolites associated with these two pathways were the most different in urine of ASD and TD children [43].

While there are several metabolic pathways that are related to the top 50 metabolites identified, about 45% of the top metabolites were connected to phenylalanine and tyrosine metabolism, fatty acid metabolism or sphingolipid metabolism. Differences in tyrosine metabolites such as decreased concentration of phenylalanine and increased concentration of p-cresol have been previously observed in studies examining the gut metabolite composition in TD and ASD children [14,44]. The role of the microbiota in this pathway is also very significant. Tyrosine metabolism pathway downregulation was observed in an ASD cohort to be associated with an increased prevalence of *Bacteroides vulgatus* while upregulation was associated with *Eggerthella lenta* [44]. Similarly, the relationship between sphingolipid metabolism and microbiome crosstalk has been suggested, and differences in short chain fatty acids of children with ASD and their TD peers have been noted [24,36].

Several metabolites related to mitochondrial metabolism and regulation such as carnitine, betaine, and adenine were also determined to have particularly high AUROC values [35,45,46,47]. Carnitine serves as the cofactor that transports long-chain fatty acids to the mitochondria matrix, and betaine plays a role in increasing mitochondrial membrane potential [45,47]. There has been considerable investigation into the relationship between ASD and mitochondrial dysfunction. It is estimated that around 4–7% of children with ASD are affected by mitochondrial disease, but it is speculated that up to 80% may have abnormalities in mitochondrial function [48,49].

Prior work has shown similar relationships between ASD fecal metabolite profiles as were observed in this study. GABA, an important neurotransmitter, was one of the metabolites identified as having a lower concentration in the ASD group prior to MTT, which is consistent with prior work [16,17]. Similarly, the fecal concentrations of free carnitine have been previously observed to be higher in children with ASD, which was also observed in this study [14]. The fecal metabolite measurements are consistent with prior work as the average indole measurements for the ASD cohort were more than twice the value of their TD counterparts at Week 0 (see Table A1) [17]. It has also been observed that fecal metabolites associated with glutamate metabolism such as 2-Keto-glutaramic acid and l-Aspartic acid were downregulated in children with ASD [44]. These metabolites were not measured in this study. Nonetheless, one of the metabolites associated with glutamate metabolism, carboxyethyl-GABA, was identified in significantly lower concentration in the ASD + GI cohort at baseline.

While there are indeed some similarities between the analysis of fecal metabolites and prior assessment of plasma samples taken from these participants, there are key distinctions. None of the metabolites identified as being utilized in the optimum multivariate models were previously identified as being significant for classification in the multivariate plasma models. The general performance of the fecal metabolites when subjected to univariate analysis had generally lower AUROC values than plasma metabolites [29]. However, despite having lower AUROC scores, multivariate analysis achieved high accuracy in distinguishing ASD and TD children. That being said, we were able to achieve greater separation using three plasma metabolites than with five fecal metabolites. This may be due to the greater homogeneity in plasma samples vs. stool samples. There have also been far more studies conducted examining plasma metabolite concentrations in individuals with ASD than studies focused on fecal samples [50,51].

Although the models were able to classify between the ASD + GI and TD cohorts with high accuracy, this study also has several limitations. The study focused exclusively on children with ASD with initially moderate to severe GI problems, which were compared to TD children with no GI issues. Therefore, assessments regarding ASD were confounded with GI problems in this analysis. ASD subgroups differentiated by variations in GI abnormalities were ignored in the analysis as subgroups were too small for a robust statistical assessment [52,53]. Furthermore, the study-cohort was not large and the ASD cohort was further split up into two different initial treatments. Future studies with a larger sample size examining cohorts with and without GI symptoms would allow for an assessment on the effectiveness of MTT in ameliorating behavioral symptoms in addition to GI related pathology.

## 5. Conclusions

This study investigated differences in fecal metabolites between a group of children diagnosed with ASD and GI symptoms and their typically developing peers with no history of GI symptoms. The univariate analysis demonstrated that individual fecal metabolites had limited potential to distinguish between ASD+GI and TD cohorts, unlike the previous study of plasma; this may be due to greater heterogeneity in stool compared to plasma. However, multivariate statistical analysis resulted in five-metabolite models that had high accuracy even after cross-validation. Both the OFM metabolite panels were shown to be capable of achieving 95% specificity and 94% sensitivity.

Following MTT, 14% of the top 50 metabolites that were found to have the greatest difference in concentration between the TD and ASD group shifted such that their distributions were significantly different eight weeks after the treatment ended. Furthermore, 94% of these metabolites reported lower AUROC following treatment, indicating diminished capacity to distinguish between the ASD and TD group. When considering a normalized average of the metabolites in the OFM models, the difference between the ASD and TD groups decreased by 82–88% at 18 weeks. These findings are similar, although less pronounced, as those determined using plasma metabolites, and both suggest that MTT resulted in shifting the metabolic profile of the ASD group towards becoming more similar to the TD group. Future work should be performed to validate the effect of MTT on fecal metabolites using a larger study cohort and a placebo arm.

## Figures and Tables

**Table 1:** The top 50 fecal metabolites (area under the receiver operator curve (AUROC) ≥ 0.66) distinguishing between ASD and typically developing (TD) groups at baseline ranked by univariate AUROC with at least 40% values above the detection limit (15 measurements). The AUROC at Week 18 is also provided. The entire list of top 165 metabolites is not shown due to space constraints (see Table A1). Either a Wilcoxon signed-rank test or a paired t-test was performed on the ASD group, comparing measurements from Week 0 to Week 18 for each of the metabolites. The *p*-value of those metabolites that significantly changed after MTT (*p*-value < 0.05) are presented. The primary associated sub-pathway for each metabolite is also provided [34].

Metabolite Rank	Metabolite	AUROC Week 0	AUROC Week 18	Pre/Post MTT *p*-Value	Associated Sub-Pathway
1	Carnitine	0.77	0.68		Carnitine Metabolism
2	Sphingosine	0.75	0.58		Sphingolipid Metabolism
3	2′-deoxyadenosine	0.75	0.59	0.02	Purine Metabolism, Adenine containing
4	Indole	0.74	0.72		Tryptophan Metabolism
5	Adenine	0.74	0.81		Purine Metabolism, Adenine containing
6	*N*-stearoyl-sphingosine (d18:1/18:0)	0.73	0.61		Sphingolipid Metabolism
7	Imidazole Propionate	0.71	0.64		Histidine Metabolism
8	10-nonadecenoate (19:1n9)	0.71	0.59	0.01	Long Chain Fatty Acid
9	p-cresol sulfate	0.71	0.53		Phenylalanine and Tyrosine Metabolism
10	Cystathionine	0.71	0.65		Methionine, Cysteine, SAM and Taurine Metabolism
11	5alpha-androstan-3beta,17alpha-diol monosulfate (1)	0.71	0.63	0.01	Steroid
12	3-(3-hydroxyphenyl)propionate	0.71	0.66		Phenylalanine and Tyrosine Metabolism
13	1-(1-enyl-oleoyl)-GPE (P-18:1)	0.71	0.87		Lysoplasmalogen
14	Deoxy-carnitine	0.71	0.58		Carnitine Metabolism
15	Gamma-glutamyl-histidine	0.71	0.68		Gamma-glutamyl Amino Acid
16	Diaminopimelate	0.70	0.62		Food Component/Plant
17	Tyramine O-sulfate	0.70	0.70		Phenylalanine and Tyrosine Metabolism
18	Gulonate	0.70	0.53		Ascorbate and Aldarate Metabolism
19	gamma-tocotrienol	0.70	0.62		Tocopherol Metabolism
20	4-hydroxyphenylacetate	0.70	0.56		Phenylalanine and Tyrosine Metabolism
21	Delta-tocopherol	0.70	0.53		Tocopherol Metabolism
22	Phenethylamine	0.69	0.55		Phenylalanine and Tyrosine Metabolism
23	Propionyl-glycine (C3)	0.69	0.61		Sphingolipid Metabolism
24	*N*-acetyl-sphingosine	0.69	0.63		Sphingolipid Metabolism
25	Betaine	0.69	0.60		Glycine, Serine and Threonine Metabolism
26	Adenosine	0.69	0.65		Purine Metabolism, Adenine containing
27	Ornithine	0.68	0.51		Urea cycle; Arginine and Proline Metabolism
28	*N*-palmitoyl-sphingosine (d18:1/16:0)	0.68	0.54	0.03	Sphingolipid Metabolism
29	Galactonate	0.68	0.67		Fructose, Mannose and Galactose Metabolism
30	N1-Methyl-2-pyridone-5-carboxamide	0.68	0.74		Nicotinate and Nicotinamide Metabolism
31	1-palmitoylglycerol (16:0)	0.68	0.54		Monoacylglycerol
32	Phosphocholine	0.67	0.59		Phospholipid Metabolism
33	Theobromine	0.67	0.62	0.02	Xanthine Metabolism
34	3,5-dihydroxybenzoic acid	0.67	0.60		Food Component/Plant
35	Hydroxyproline	0.67	0.56		Urea cycle; Arginine and Proline Metabolism
36	l-urobilin	0.67	0.60	0.02	Hemoglobin and Porphyrin Metabolism
37	carboxyethyl-GABA	0.67	0.55		Glutamate Metabolism
38	oxalate (ethane-dioate)	0.67	0.53		Ascorbate and Aldarate Metabolism
39	Palmitoyl-carnitine (C16)	0.67	0.53		Fatty Acid Metabolism(Acyl Carnitine)
40	Copro-stanol	0.67	0.60		Sterol
41	Saccharopine	0.66	0.57		Lysine Metabolism
42	5-hydroxylysine	0.66	0.53		Lysine Metabolism
43	Stearoyl-carnitine (C18)	0.66	0.58		Fatty Acid Metabolism(Acyl Carnitine)
44	Biliverdin	0.66	0.60		Hemoglobin and Porphyrin Metabolism
45	3-(4-hydroxyphenyl)lactate (HPLA)	0.66	0.59		Phenylalanine and Tyrosine Metabolism
46	Carnosine	0.66	0.57		Dipeptide Derivative
47	10-hydroxystearate	0.66	0.64	0.01	Fatty Acid, Monohydroxy
48	Pentadecanoate (15:0)	0.66	0.55		Long Chain Fatty Acid
49	Hexadecanedioate (C16)	0.66	0.60		Fatty Acid, Dicarboxylate
50	Sphinganine	0.66	0.54		Sphingolipid Metabolism

**Table 2:** Fitting and cross-validation results for the best combinations of two, three, four, and five metabolites used as part of Fisher Discriminant Analysis (FDA). The cross-validated true positive rate (TPR) and true negative rate (TNR) are shown for classification thresholds associated with different values of β calculated from the fitted probability density functions (PDFs). The results for two distinct 5-metabolite models are presented as they were able to achieve the same accuracy following cross-validation. The notable TPRs and TNRs are highlighted for the 5-metabolite models.

Number of Metabolites	Metabolite Combination	Fitted AUROC	Cross-Validated Results		
			β	TPR	TNR
2	Carnitine 2′deoxyadenosine	0.88	0.01	1.00	0.20
			0.05	1.00	0.35
			0.10	1.00	0.50
			0.20	0.89	0.75
3	Adenosine theobromine hydroxyproline	0.93	0.01	1.00	0.30
			0.05	0.94	0.55
			0.10	0.94	0.75
			0.20	0.72	0.85
4	indole	0.98	0.01	0.94	0.35
	1-(1-enyl-oleoyl)-GPE (P-18:1)		0.05	0.89	0.60
	Hydroxyproline		0.10	0.83	0.60
	Carnosine		0.20	0.83	0.80
5	Imidazole Propionate	1.00	0.01	1.00	0.85
	Hydroxyproline		0.05	0.94	0.95
	Theobromine		0.10	0.89	0.95
	2-hydroxy-3-methylvalerate Adenosine		0.20	0.78	0.95
5	Imidazole Propionate	1.00	0.01	1.00	0.85
	Hydroxyproline		0.05	0.94	0.95
	Theobromine		0.10	0.89	0.95
	2-hydroxy-3-methylvalerate		0.20	0.78	0.95
	Indole				

**Table 3:** The correlation coefficients between the optimized fecal model-indole/adenosine (OFM-I/A) metabolites and top metabolites 50 metabolites are examined and presented in order of their AUROC. Only those correlations that are significant (*p*-value < 0.05) are presented.

Metabolite	Correlation Coefficient	*p*-Value
*Indole*		
Carnitine	0.67	<0.001
Indole-lactate	0.56	<0.001
Saccharopine	0.42	0.007
Stearoyl-carnitine	0.39	0.015
3-(3-hydroxyphenyl)propionate	0.33	0.043
Oxalate	0.33	0.043
*Imidazole Propionate*		
Galactonate	0.78	<0.001
Gulonate	0.76	<0.001
Palmitoyl-carnitine	0.72	<0.001
Saccharopine	0.7	<0.001
Phosphocholine	0.69	<0.001
Cystathionine	0.62	<0.001
Phenethylamine	0.61	<0.001
Betaine	0.61	<0.001
3-(4-hydroxyphenyl)lactate	0.6	<0.001
*N*-propionyl-methionine	0.58	<0.001
*N*-palmitoyl-sphingosine	0.41	0.011
3,5-dihydroxybenzoic	0.4	0.014
3-(3-hydroxyphenyl)propionate	0.39	0.017
Stearoyl-carnitine	0.38	0.018
1-palmitoylglycerol	0.37	0.023
Gamma-glutamyl-histidine	0.36	0.027
Biliverdin	0.34	0.037
Carnitine	0.32	0.048
*Adenosine*		
Adenine	0.74	<0.001
2′-deoxyadenosine	0.54	<0.001
5-hydroxylysine	0.36	0.0254
Hydroxyproline	0.36	0.0256
5-hydroxylysine	0.36	0.0254
1-(1-enyl-oleoyl)-GPE	0.34	0.0366
Theobromine **		
*None*		
*Hydroxyproline*		
2-hydroxy-3-methylvalerate ***	0.61	<0.001
delta-tocopherol	0.41	0.011
2′-deoxyadenosine	0.38	0.017
Adenosine	0.36	0.026
Copro-stanol	0.36	0.026
5alpha-androstan-3beta,17alpha-diol	0.35	0.030
p-cresol	−0.32	0.050
Betaine	−0.33	0.043
Oxalate	−0.35	0.031
*N*-palmitoyl-sphingosine	−0.37	0.023
2-hydroxy-3-methylvalerate ***		
Gulonate	0.81	<0.001
Imidazole propionate	0.79	<0.001
Galactonate	0.78	<0.001
Phosphocholine	0.75	<0.001
5-hydroxylysine	0.72	<0.001
Hydroxyproline	0.61	<0.001
Betaine	0.6	<0.001
Phenethylamine	0.59	<0.001
1-(1-enyl-oleoyl)-GPE (P-18:1)	0.55	<0.001
Cystathionine	0.53	<0.001
1-palmitoylglycerol (16:0)	0.51	<0.001
Biliverdin	0.51	0.001
Propionyl-glycine (C3)	0.45	0.005
3-(4-hydroxyphenyl)lactate (HPLA)	0.43	0.008
3-(3-hydroxyphenyl)propionate	0.34	0.039
Delta-tocopherol	−0.33	0.042
Copro-stanol	−0.35	0.033

** Theobromine was not found to be significantly correlated with any of the top 50 metabolites. *** 2-hydroxy-3-methylvalerate is not among the top 50 metabolites as ranked by AUROC but present in both OFM-I/A panels.

**Table 4:** Change in the difference between OFM-I/A metabolites measured in the TD and ASD cohort over the course of the study. The discriminant score was calculated by first taking the absolute value of the difference between measurements at each time point and the median of the TD group, then normalizing the difference by the standard deviation of the TD Week 0 measurements, and then adding the normalized absolute difference for each of the five metabolites. The background color distinguished the individual metabolites from the multivariate models.

	ASD Week 0	ASD Week 3	ASD Week 10	ASD Week 18	TD Week 0
Imidazole Propionate (25th/75th percentile)	0.37 (0.19, 3.38)	0.55 (0.08, 8.57)	0.29 (0.08, 0.80)	0.14 (0.03, 0.46)	0.12 (0.09, 0.29)
Hydroxyproline (25th/75th percentile)	0.96 (0.72, 4.83)	1.06 (0.42, 3.67)	1.27 (0.24, 3.34)	0.80 (0.54, 3.60)	0.60 (0.29, 1.20)
Theobromine (25th/75th percentile)	0.89 (0.47, 2.38)	0.47 (0.47, 1.68)	0.47 (0.16, 0.47)	0.47 (0.43, 0.47)	0.46 (0.34, 0.64)
2-hydroxy-3-methylvalerate (25th/75th percentile)	0.53 (0.43, 0.75)	0.43 (0.18, 0.56)	0.34 (0.06, 0.50)	0.52 (0.21, 0.63)	0.44 (0.21, 0.61)
Indole (25th/75th percentile)	1.13 (0.25, 1.83)	0.66 (0.32, 1.75)	0.85 (0.18, 1.86)	0.86 (0.26, 1.52)	0.39 (0.15, 0.59)
Adenosine (25th/75th percentile)	0.67 (0.36, 0.88)	0.77 (0.50, 1.01)	0.73 (0.47, 0.90)	0.57 (0.26, 0.86)	0.40 (0.18, 0.86)
OFM-I Median discriminant score (25th/75th percentile)	3.90 (2.33, 5.72)	1.90 (0.72, 9.52)	1.84 (0.90, 3.71)	1.73 (0.71, 2.62)	0.46 (0.21, 1.35)
Type II error	5%	53%	50%	56%	-
OFM-A Median discriminant score (25th/75th percentile)	3.51 (2.28, 5.73)	2.87 (1.13, 9.43)	2.18 (1.07, 4.18)	1.36 (0.54, 2.44)	0.62 (0.35, 1.05)
Type II error	5%	53%	39%	56%	-

## Appendix A

**Table A1:** Univariate assessment of all metabolites with an AUROC greater than 0.60. The AUROC is provided at Week 0 for the TD and ASD cohort as well as average ultrahigh performance liquid chromatography-tandem mass spectroscopy (UHPLC-MS/MS) signal intensity measurements for both cohorts. The *p*-value for the significance between the ASD and TD cohorts at Week 0 and between the ASD cohort at Week 0 and Week 18 are also shown. Finally, the results of leave-n-out (*n* = 1–3) cross-validation are provided.

Metabolite	Week 0 AUROC	Week 18 AUROC	TD Mean Week 0	ASD Mean Week 0	Week 0 vs. Week 18 ASD *p*-Value	Week 0 ASD vs. TD Un-Adjusted *p*-Value	Leave-1-Out	Leave 2-Out	Leave 3-Out
carnitine	0.77	0.68	9.81 × 10^6^	3.35 × 10^7^	7.64 × 10^−1^	4.78 × 10^−3^	0.00	0.00	0.00
sphingosine	0.75	0.58	7.18 × 10^6^	2.13 × 10^7^	1.10 × 10^−1^	8.15 × 10^−3^	0.00	0.00	0.00
2′-deoxyadenosine	0.75	0.59	8.08 × 10^5^	2.32 × 10^5^	1.83 × 10^−2^	9.59 × 10^−3^	0.00	0.00	0.00
indole	0.74	0.72	4.74 × 10^5^	1.05 × 10^6^	5.80 × 10^−1^	1.04 × 10^−2^	0.00	0.00	0.00
adenine	0.74	0.81	6.70 × 10^6^	2.91 × 10^6^	5.17 × 10^−1^	1.35 × 10^−2^	0.00	0.00	0.03
*N*-stearoyl-sphingosine	0.73	0.61	1.01 × 10^6^	4.05 × 10^6^	1.03 × 10^−1^	1.40 × 10^−2^	0.00	0.00	0.04
imidazole	0.71	0.64	1.02 × 10^7^	3.95 × 10^7^	1.50 × 10^−1^	1.83 × 10^−2^	0.00	0.00	0.13
10-nonadecenoate	0.71	0.59	2.42 × 10^6^	5.49 × 10^6^	1.19 × 10^−2^	2.18 × 10^−2^	0.00	0.01	0.25
p-cresol	0.71	0.53	1.55 × 10^6^	4.00 × 10^6^	6.42 × 10^−2^	2.53 × 10^−2^	0.00	0.16	0.27
cystathionine	0.71	0.65	6.07 × 10^4^	9.12 × 10^4^	5.98 × 10^−1^	2.53 × 10^−2^	0.00	0.17	0.25
5alpha-androstan-3beta,17alpha-diol	0.71	0.63	9.86 × 10^4^	6.65 × 10^4^	4.50 × 10^−3^	2.55 × 10^−2^	0.00	0.31	0.32
3-(3-hydroxyphenyl)propionate	0.71	0.66	2.24 × 10^7^	2.67 × 10^7^	8.62 × 10^−1^	2.73 × 10^−2^	0.00	0.24	0.30
1-(1-enyl-oleoyl)-GPE	0.71	0.87	3.31 × 10^5^	2.57 × 10^5^	2.47 × 10^−1^	2.81 × 10^−2^	0.00	0.24	0.29
gamma-glutamyl-histidine	0.71	0.58	1.90 × 10^5^	1.95 × 10^5^	1.83 × 10^−1^	2.94 × 10^−2^	0.00	0.25	0.38
Deoxy-carnitine	0.71	0.68	2.82 × 10^8^	1.09 × 10^9^	6.46 × 10^−1^	2.94 × 10^−2^	0.00	0.26	0.42
diaminopimelate	0.70	0.62	8.57 × 10^5^	1.14 × 10^6^	7.16 × 10^−1^	3.11 × 10^−2^	0.00	0.32	0.40
tyramine	0.70	0.70	2.17 × 10^7^	8.33 × 10^6^	7.86 × 10^−1^	3.17 × 10^−2^	0.21	0.27	0.36
gulonate	0.70	0.53	2.43 × 10^5^	1.52 × 10^6^	9.12 × 10^−2^	3.40 × 10^−2^	0.11	0.42	0.46
gamma-tocotrienol	0.70	0.62	5.04 × 10^6^	3.24 × 10^6^	3.51 × 10^−1^	3.49 × 10^−2^	0.26	0.42	0.46
4-hydroxyphenylacetate	0.70	0.56	9.97 × 10^5^	2.75 × 10^6^	2.75 × 10^−1^	3.66 × 10^−2^	0.32	0.42	0.44
delta-tocopherol	0.70	0.53	2.50 × 10^6^	1.62 × 10^6^	7.38 × 10^−2^	3.85 × 10^−2^	0.42	0.49	0.48
phenethylamine	0.69	0.55	4.35 × 10^5^	9.09 × 10^5^	6.42 × 10^−2^	3.93 × 10^−2^	0.39	0.45	0.49
Propionyl-glycine	0.69	0.61	1.56 × 10^5^	3.92 × 10^5^	6.09 × 10^−1^	3.93 × 10^−2^	0.47	0.51	0.53
*N*-acetyl-sphingosine	0.69	0.63	9.84 × 10^4^	2.63 × 10^5^	3.33 × 10^−1^	4.22 × 10^−2^	0.45	0.51	0.47
betaine	0.69	0.60	3.39 × 10^6^	5.05 × 10^6^	5.37 × 10^−1^	4.55 × 10^−2^	0.63	0.50	0.47
adenosine	0.69	0.65	5.81 × 10^5^	3.41 × 10^5^	5.58 × 10^−1^	4.84 × 10^−2^	0.50	0.59	0.60
ornithine	0.68	0.51	2.05 × 10^7^	2.74 × 10^7^	6.89 × 10^−2^	5.19 × 10^−2^	0.71	0.61	0.65
*N*-palmitoyl-sphingosine	0.68	0.54	3.01 × 10^6^	1.04 × 10^7^	2.57 × 10^−2^	5.33 × 10^−2^	1.00	1.00	0.67
galactonate	0.68	0.67	4.63 × 10^5^	2.05 × 10^6^	7.63 × 10^−1^	5.37 × 10^−2^	0.66	0.62	0.67
N1-Methyl-2-pyridone-5-carboxamide	0.68	0.74	1.63 × 10^5^	4.37 × 10^5^	7.62 × 10^−1^	5.44 × 10^−2^	1.00	1.00	0.69
1-palmitoylglycerol	0.68	0.54	1.08 × 10^7^	1.55 × 10^7^	1.69 × 10^−1^	5.72 × 10^−2^	1.00	1.00	0.71
phosphocholine	0.67	0.59	3.01 × 10^5^	1.25 × 10^7^	5.36 × 10^−2^	5.93 × 10^−2^	0.79	0.72	0.72
theobromine	0.67	0.62	2.60 × 10^6^	5.81 × 10^6^	1.56 × 10^−2^	5.93 × 10^−2^	0.76	0.70	0.72
hydroxyproline	0.67	0.60	2.96 × 10^6^	9.08 × 10^6^	6.92 × 10^−1^	6.10 × 10^−2^	0.79	0.73	0.74
l-urobilin	0.67	0.56	4.11 × 10^7^	3.08 × 10^7^	1.55 × 10^−2^	6.35 × 10^−2^	0.71	0.80	0.73
3,5-dihydroxybenzoic	0.67	0.60	1.61 × 10^5^	9.06 × 10^4^	3.29 × 10^−1^	6.52 × 10^−2^	1.00	1.00	0.95
carboxyethyl-GABA	0.67	0.55	1.52 × 10^7^	1.01 × 10^7^	2.00 × 10^−1^	6.52 × 10^−2^	1.00	1.00	0.95
oxalate	0.67	0.53	1.03 × 10^6^	2.11 × 10^6^	7.91 × 10^−2^	6.58 × 10^−2^	0.74	0.80	0.78
Palmitoyl-carnitine	0.67	0.53	4.93 × 10^5^	4.68 × 10^6^	5.18 × 10^−2^	6.77 × 10^−2^	0.79	0.79	0.79
Copro-stanol	0.67	0.60	2.21 × 10^6^	1.31 × 10^6^	3.17 × 10^−1^	6.86 × 10^−2^	0.87	0.79	0.79
5-hydroxylysine	0.66	0.57	1.03 × 10^6^	2.53 × 10^6^	3.35 × 10^−1^	6.99 × 10^−2^	0.82	0.82	0.80
Saccharopine	0.66	0.53	1.50 × 10^6^	4.10 × 10^5^	9.49 × 10^−2^	7.22 × 10^−2^	0.89	0.82	0.79
3-(4-hydroxyphenyl)lactate	0.66	0.58	1.56 × 10^6^	1.88 × 10^6^	2.37 × 10^−2^	7.22 × 10^−2^	0.82	0.82	0.81
Stearoyl-carnitine	0.66	0.60	1.65 × 10^6^	4.06 × 10^6^	1.03 × 10^−1^	7.69 × 10^−2^	0.92	0.85	0.82
biliverdin	0.66	0.59	1.29 × 10^5^	2.88 × 10^5^	3.67 × 10^−1^	7.69 × 10^−2^	0.89	0.85	0.82
carnosine	0.66	0.57	8.48 × 10^4^	8.23 × 10^4^	3.44 × 10^−1^	8.31 × 10^−2^	0.87	0.91	0.85
pentadecanoate	0.66	0.64	2.40 × 10^8^	1.97 × 10^8^	8.62 × 10^−1^	8.44 × 10^−2^	0.87	0.87	0.87
hexadecanedioate	0.66	0.55	4.54 × 10^5^	4.28 × 10^5^	1.25 × 10^−1^	8.72 × 10^−2^	0.89	0.90	0.87
10-hydroxystearate	0.66	0.60	1.16 × 10^9^	1.93 × 10^9^	8.25 × 10^−3^	9.27 × 10^−2^	0.95	0.92	0.88
Sphinganine	0.66	0.54	1.67 × 10^7^	2.34 × 10^7^	3.19 × 10^−1^	9.28 × 10^−2^	0.89	0.91	0.89
trigonelline	0.66	0.81	3.08 × 10^6^	6.92 × 10^6^	1.41 × 10^−1^	9.28 × 10^−2^	0.92	0.92	0.88
Indole-lactate	0.66	0.64	4.74 × 10^5^	1.80 × 10^6^	8.80 × 10^−3^	9.46 × 10^−2^	1.00	1.00	0.87
Dihomo-linoleate	0.65	0.58	1.11 × 10^7^	2.92 × 10^7^	7.38 × 10^−2^	9.76 × 10^−2^	1.00	1.00	1.00
Phyto-sphingosine	0.65	0.60	8.22 × 10^5^	1.25 × 10^6^	5.58 × 10^−1^	9.77 × 10^−2^	1.00	0.95	0.90
gamma-tocopherol/beta-tocopherol	0.65	0.51	5.43 × 10^7^	4.05 × 10^7^	1.03 × 10^−1^	9.86 × 10^−2^	0.92	0.91	0.89
acesulfame	0.65	0.59	3.59 × 10^4^	7.24 × 10^5^	6.27 × 10^−1^	9.86 × 10^−2^	0.92	0.92	0.90
*N*-methyl-pipecolate	0.65	0.63	4.88 × 10^6^	9.71 × 10^6^	7.16 × 10^−1^	9.86 × 10^−2^	0.97	0.94	0.88
2-methylserine	0.65	0.66	9.22 × 10^5^	7.05 × 10^5^	7.16 × 10^−1^	9.86 × 10^−2^	0.97	0.93	0.90
2-aminobutyrate	0.65	0.61	1.27 × 10^7^	2.65 × 10^7^	4.02 × 10^−1^	1.02 × 10^−1^	0.97	0.94	0.92
*N*-palmitoyl-sphinganine	0.65	0.64	1.99 × 10^6^	4.43 × 10^6^	1.55 × 10^−2^	1.02 × 10^−1^	0.95	0.94	0.91
caffeate	0.65	0.60	4.23 × 10^5^	4.23 × 10^5^	5.19 × 10^−1^	1.06 × 10^−1^	0.97	0.95	0.94
piperine	0.65	0.59	2.41 × 10^7^	1.59 × 10^7^	1.96 × 10^−2^	1.11 × 10^−1^	1.00	0.96	0.93
*N*-propionyl-methionine	0.65	0.65	1.55 × 10^5^	3.58 × 10^5^	8.87 × 10^−1^	1.11 × 10^−1^	1.00	0.96	0.93
alpha-CEHC	0.65	0.50	1.02 × 10^5^	5.68 × 10^5^	6.53 × 10^−2^	1.11 × 10^−1^	0.97	0.96	0.93
5-aminovalerate	0.65	0.55	3.29 × 10^7^	1.16 × 10^8^	4.57 × 10^−1^	1.11 × 10^−1^	1.00	1.00	0.94
2-aminophenol	0.65	0.56	7.90 × 10^5^	6.54 × 10^5^	4.02 × 10^−1^	1.17 × 10^−1^	1.00	1.00	0.93
*O*-sulfo-l-tyrosine	0.65	0.50	9.04 × 10^4^	3.24 × 10^5^	1.26 × 10^−1^	1.17 × 10^−1^	0.97	0.95	0.93
*N*-acetyl-valine	0.64	0.69	1.99 × 10^5^	9.36 × 10^5^	9.50 × 10^−1^	1.18 × 10^−1^	1.00	0.96	0.94
norvaline	0.64	0.52	4.20 × 10^6^	1.65 × 10^7^	2.05 × 10^−1^	1.18 × 10^−1^	1.00	0.96	0.94
tryptamine	0.64	0.93	3.40 × 10^5^	1.63 × 10^6^	1.19 × 10^−2^	1.18 × 10^−1^	1.00	0.96	0.94
myristate	0.64	0.73	3.65 × 10^8^	3.05 × 10^8^	3.04 × 10^−1^	1.18 × 10^−1^	1.00	1.00	0.94
Eicosenoate	0.64	0.59	5.55 × 10^7^	1.30 × 10^8^	7.91 × 10^−2^	1.19 × 10^−1^	1.00	1.00	0.95
cholesterol	0.64	0.56	3.70 × 10^6^	1.35 × 10^7^	3.67 × 10^−1^	1.20 × 10^−1^	1.00	0.98	0.95
3-ureidopropionate	0.64	0.58	6.14 × 10^5^	3.26 × 10^5^	6.92 × 10^−1^	1.24 × 10^−1^	1.00	0.95	0.93
diglycerol	0.64	0.60	3.13 × 10^6^	1.50 × 10^6^	5.17 × 10^−1^	1.25 × 10^−1^	1.00	0.97	0.95
*N*-acetylneuraminate	0.64	0.52	4.66 × 10^6^	9.65 × 10^6^	1.25 × 10^−1^	1.25 × 10^−1^	1.00	0.96	0.94
Succinyl-carnitine	0.64	0.65	6.00 × 10^5^	3.56 × 10^6^	8.87 × 10^−1^	1.29 × 10^−1^	1.00	0.97	0.97
2′-deoxyinosine	0.64	0.76	4.88 × 10^6^	7.84 × 10^6^	1.69 × 10^−1^	1.30 × 10^−1^	1.00	0.99	0.97
3-aminoisobutyrate	0.64	0.54	2.82 × 10^5^	3.10 × 10^6^	3.41 × 10^−1^	1.32 × 10^−1^	1.00	0.99	0.96
d-urobilin	0.64	0.54	7.74 × 10^5^	2.68 × 10^6^	6.42 × 10^−2^	1.36 × 10^−1^	1.00	0.99	0.97
1-methylnicotinamide	0.64	0.68	1.02 × 10^5^	2.95 × 10^5^	5.58 × 10^−1^	1.36 × 10^−1^	1.00	0.98	0.97
*N*-acetyl-alanine	0.64	0.58	3.06 × 10^6^	3.98 × 10^6^	3.84 × 10^−1^	1.38 × 10^−1^	1.00	1.00	0.98
aspartate	0.64	0.54	1.90 × 10^8^	2.44 × 10^8^	1.03 × 10^−1^	1.40 × 10^−1^	1.00	0.98	0.96
trans-urocanate	0.64	0.50	4.71 × 10^7^	3.96 × 10^7^	1.17 × 10^−1^	1.40 × 10^−1^	1.00	0.98	0.97
3-carboxyadipate	0.64	0.66	8.99 × 10^6^	1.47 × 10^7^	5.58 × 10^−1^	1.40 × 10^−1^	1.00	0.98	0.96
1,7-dimethylurate	0.64	0.60	8.91 × 10^5^	2.31 × 10^6^	2.87 × 10^−2^	1.40 × 10^−1^	1.00	0.98	0.97
2-piperidinone	0.64	0.64	2.17 × 10^7^	3.82 × 10^7^	9.37 × 10^−1^	1.40 × 10^−1^	1.00	1.00	0.97
pheophorbide	0.64	0.61	4.12 × 10^6^	4.78 × 10^5^	6.94 × 10^−1^	1.41 × 10^−1^	1.00	1.00	1.00
acisoga	0.63	0.63	3.52 × 10^5^	6.39 × 10^5^	6.92 × 10^−1^	1.41 × 10^−1^	1.00	1.00	1.00
sulfate	0.63	0.55	6.68 × 10^6^	1.65 × 10^7^	1.10 × 10^−1^	1.41 × 10^−1^	1.00	1.00	1.00
1-(1-enyl-palmitoyl)-GPE	0.63	0.79	8.53 × 10^5^	7.57 × 10^5^	5.11 × 10^−2^	1.41 × 10^−1^	0.97	0.96	0.95
histidine	0.63	0.55	9.65 × 10^7^	1.10 × 10^8^	2.00 × 10^−1^	1.43 × 10^−1^	1.00	1.00	0.97
Maltotetraose	0.63	0.53	5.10 × 10^5^	6.33 × 10^5^	2.66 × 10^−1^	1.48 × 10^−1^	1.00	1.00	0.98
maltose	0.63	0.63	7.98 × 10^6^	5.18 × 10^6^	9.62 × 10^−1^	1.48 × 10^−1^	1.00	1.00	0.98
2-methylcitrate/homocitrate	0.63	0.66	6.24 × 10^5^	9.91 × 10^5^	9.87 × 10^−1^	1.48 × 10^−1^	1.00	0.98	0.97
trimethylamine	0.63	0.55	3.98 × 10^5^	1.53 × 10^6^	3.35 × 10^−1^	1.48 × 10^−1^	1.00	0.99	0.97
linoleoyl-linolenoyl-glycerol	0.63	0.53	8.05 × 10^6^	2.50 × 10^6^	1.33 × 10^−1^	1.48 × 10^−1^	1.00	0.99	0.97
thymidine	0.63	0.66	5.10 × 10^6^	7.32 × 10^6^	9.87 × 10^−1^	1.52 × 10^−1^	1.00	0.98	0.98
Pyri-doxate	0.63	0.52	1.40 × 10^7^	1.76 × 10^7^	1.69 × 10^−1^	1.52 × 10^−1^	1.00	0.98	0.98
sarcosine	0.63	0.66	2.59 × 10^5^	6.59 × 10^5^	6.12 × 10^−1^	1.56 × 10^−1^	0.97	0.95	0.94
2-hydroxy-3-methylvalerate	0.63	0.54	1.27 × 10^6^	1.74 × 10^6^	5.06 × 10^−1^	1.56 × 10^−1^	1.00	0.99	0.98
gamma-glutamyl-phenylalanine	0.63	0.61	2.27 × 10^5^	2.94 × 10^5^	9.50 × 10^−1^	1.56 × 10^−1^	1.00	0.99	0.98
Linolenate	0.63	0.59	1.98 × 10^8^	9.80 × 10^7^	9.87 × 10^−1^	1.56 × 10^−1^	1.00	0.98	0.97
3-hydroxy-3-methylglutarate	0.63	0.59	1.40 × 10^7^	1.99 × 10^7^	5.17 × 10^−1^	1.56 × 10^−1^	1.00	0.98	0.97
2′-deoxyuridine	0.63	0.69	2.81 × 10^6^	3.79 × 10^6^	6.46 × 10^−1^	1.56 × 10^−1^	1.00	1.00	0.98
Quino-linate	0.63	0.52	4.89 × 10^4^	1.15 × 10^5^	9.77 × 10^−2^	1.57 × 10^−1^	1.00	1.00	0.98
I-urobilinogen	0.63	0.60	1.77 × 10^6^	1.02 × 10^7^	3.91 × 10^−2^	1.65 × 10^−1^	1.00	1.00	0.98
*N*-acetyl-cadaverine	0.63	0.61	4.95 × 10^7^	1.14 × 10^8^	7.64 × 10^−1^	1.65 × 10^−1^	1.00	1.00	0.99
7-ketolithocholate	0.63	0.51	4.53 × 10^6^	1.32 × 10^6^	1.92 × 10^−1^	1.69 × 10^−1^	1.00	1.00	0.98
Carboxy-ibuprofen	0.63	0.53	1.47 × 10^6^	1.75 × 10^4^	1.48 × 10^−1^	1.74 × 10^−1^	1.00	0.99	0.98
Phenyl-lactate	0.62	0.57	1.83 × 10^6^	1.83 × 10^6^	4.13 × 10^−2^	1.74 × 10^−1^	1.00	0.99	0.98
kynurenate	0.62	0.59	1.30 × 10^6^	9.08 × 10^5^	9.05 × 10^−2^	1.74 × 10^−1^	1.00	0.99	0.98
citrate	0.62	0.58	1.29 × 10^6^	2.64 × 10^6^	5.91 × 10^−1^	1.74 × 10^−1^	1.00	1.00	0.99
5alpha-androstan-3beta,17beta-diol	0.62	0.53	1.85 × 10^5^	7.81 × 10^4^	1.06 × 10^−1^	1.74 × 10^−1^	1.00	1.00	0.99
Octadecane-dioate	0.62	0.66	1.10 × 10^6^	8.50 × 10^5^	2.67 × 10^−2^	1.74 × 10^−1^	1.00	0.99	0.98
Oleoyl-carnitine	0.62	0.54	4.89 × 10^5^	4.10 × 10^6^	1.35 × 10^−1^	1.74 × 10^−1^	1.00	0.99	0.98
4-androsten-3alpha,17alpha-diol	0.62	0.50	2.30 × 10^5^	7.59 × 10^4^	2.02 × 10^−1^	1.83 × 10^−1^	1.00	1.00	0.99
8-hydroxyguanine	0.62	0.55	1.15 × 10^5^	1.50 × 10^5^	1.58 × 10^−1^	1.83 × 10^−1^	1.00	1.00	0.98
skatol	0.62	0.50	5.08 × 10^5^	3.96 × 10^5^	1.71 × 10^−1^	1.83 × 10^−1^	1.00	1.00	0.99
Ethyl-malonate	0.62	0.66	5.50 × 10^6^	6.23 × 10^6^	4.20 × 10^−1^	1.83 × 10^−1^	1.00	1.00	0.99
lactate	0.62	0.52	2.39 × 10^6^	3.29 × 10^6^	1.41 × 10^−1^	1.83 × 10^−1^	1.00	1.00	0.99
N6-carboxymethyllysine	0.62	0.60	2.90 × 10^6^	3.69 × 10^6^	5.37 × 10^−1^	1.86 × 10^−1^	1.00	1.00	1.00
Nervonate	0.62	0.53	6.63 × 10^6^	1.61 × 10^7^	3.67 × 10^−1^	1.86 × 10^−1^	1.00	1.00	1.00
inosine	0.62	0.57	3.45 × 10^6^	6.39 × 10^6^	7.16 × 10^−1^	1.86 × 10^−1^	1.00	1.00	0.99
Docosapentaenoate	0.61	0.55	3.98 × 10^6^	1.35 × 10^7^	7.90 × 10^−2^	1.89 × 10^−1^	1.00	1.00	0.99
Acetyl-carnitine	0.61	0.54	7.50 × 10^5^	8.06 × 10^6^	4.77 × 10^−1^	1.89 × 10^−1^	1.00	1.00	0.98
*N*-methyl-GABA	0.61	0.59	5.35 × 10^6^	6.36 × 10^6^	9.35 × 10^−1^	1.93 × 10^−1^	1.00	1.00	0.99
2-aminoadipate	0.61	0.66	1.11 × 10^6^	1.27 × 10^6^	7.40 × 10^−1^	1.95 × 10^−1^	1.00	1.00	0.99
*N*-methyl-phenylalanine	0.61	0.57	1.65 × 10^6^	1.25 × 10^6^	7.38 × 10^−2^	1.95 × 10^−1^	1.00	1.00	0.99
cystine	0.61	0.59	8.29 × 10^4^	1.14 × 10^5^	9.49 × 10^−3^	1.98 × 10^−1^	1.00	1.00	1.00
3-hydroxystearate	0.61	0.54	6.21 × 10^6^	4.61 × 10^6^	7.88 × 10^−1^	2.00 × 10^−1^	1.00	1.00	0.99
gluconate	0.61	0.51	1.08 × 10^6^	3.21 × 10^6^	6.90 × 10^−1^	2.02 × 10^−1^	1.00	1.00	1.00
diacylglycerol	0.61	0.51	6.93 × 10^5^	2.55 × 10^5^	3.02 × 10^−1^	2.03 × 10^−1^	1.00	1.00	0.99
dipicolinate	0.61	0.51	4.99 × 10^5^	4.94 × 10^5^	2.46 × 10^−1^	2.03 × 10^−1^	1.00	1.00	0.99
quinate	0.61	0.61	9.58 × 10^6^	2.75 × 10^7^	9.37 × 10^−1^	2.03 × 10^−1^	1.00	1.00	0.99
*O*-acetyl-homoserine	0.61	0.52	4.56 × 10^5^	3.96 × 10^5^	4.20 × 10^−1^	2.14 × 10^−1^	1.00	1.00	0.99
glutamate,	0.61	0.51	2.42 × 10^6^	2.03 × 10^6^	2.75 × 10^−1^	2.14 × 10^−1^	1.00	1.00	0.99
6-hydroxynicotinate	0.61	0.58	1.82 × 10^6^	8.38 × 10^5^	3.67 × 10^−1^	2.18 × 10^−1^	1.00	1.00	1.00
pyridoxine	0.61	0.61	1.57 × 10^6^	2.91 × 10^6^	5.47 × 10^−2^	2.18 × 10^−1^	1.00	1.00	1.00
2,4,6-trihydroxybenzoate	0.61	0.55	3.58 × 10^4^	5.36 × 10^5^	6.48 × 10^−1^	2.20 × 10^−1^	1.00	1.00	1.00
theophylline	0.61	0.57	1.46 × 10^5^	8.09 × 10^4^	6.37 × 10^−1^	2.21 × 10^−1^	1.00	1.00	1.00
1-methylimidazoleacetate	0.61	0.53	2.51 × 10^6^	2.47 × 10^6^	1.17 × 10^−1^	2.21 × 10^−1^	1.00	1.00	1.00
1-methylhistamine	0.61	0.55	1.63 × 10^5^	5.62 × 10^5^	4.69 × 10^−1^	2.25 × 10^−1^	1.00	1.00	0.99
phenylacetate	0.61	0.58	1.69 × 10^7^	2.48 × 10^7^	7.16 × 10^−1^	2.25 × 10^−1^	1.00	1.00	0.99
4-hydroxyphenylpyruvate	0.61	0.57	5.26 × 10^5^	5.38 × 10^5^	6.69 × 10^−1^	2.25 × 10^−1^	1.00	1.00	1.00
cysteine	0.61	0.61	3.35 × 10^6^	4.04 × 10^6^	8.62 × 10^−1^	2.25 × 10^−1^	1.00	1.00	1.00
*N*-acetylcysteine	0.61	0.51	3.09 × 10^5^	3.95 × 10^5^	2.23 × 10^−1^	2.25 × 10^−1^	1.00	1.00	0.99
13-methylmyristate	0.61	0.72	3.09 × 10^5^	3.95 × 10^5^	2.89 × 10^−1^	2.27 × 10^−1^	1.00	1.00	1.00
AMP	0.61	0.52	3.56 × 10^8^	3.17 × 10^8^	2.44 × 10^−1^	2.29 × 10^−1^	1.00	1.00	1.00
2′-deoxyguanosine	0.61	0.75	1.20 × 10^5^	5.63 × 10^4^	1.79 × 10^−1^	2.36 × 10^−1^	1.00	1.00	1.00
5-methyluridine	0.61	0.64	2.32 × 10^6^	3.59 × 10^6^	8.62 × 10^−1^	2.36 × 10^−1^	1.00	1.00	1.00
1,3-dimethylurate	0.61	0.58	7.67 × 10^5^	9.16 × 10^5^	5.20 × 10^−2^	2.36 × 10^−1^	1.00	1.00	1.00
adrenate	0.60	0.64	3.54 × 10^4^	2.94 × 10^4^	9.61 × 10^−1^	2.45 × 10^−1^	1.00	1.00	0.99
tryptophan	0.60	0.51	1.44 × 10^7^	1.86 × 10^7^	3.67 × 10^−1^	2.47 × 10^−1^	1.00	1.00	1.00
dimethylarginine	0.60	0.52	2.18 × 10^6^	6.82 × 10^5^	4.77 × 10^−1^	2.47 × 10^−1^	1.00	1.00	1.00
4-acetamidobutanoate	0.60	0.50	7.64 × 10^7^	8.86 × 10^7^	3.34 × 10^−1^	2.47 × 10^−1^	1.00	1.00	1.00
gamma-glutamyl-glutamine	0.60	0.52	3.61 × 10^6^	5.50 × 10^6^	3.51 × 10^−1^	2.48 × 10^−1^	1.00	1.00	1.00
caproate	0.60	0.72	1.31 × 10^6^	1.72 × 10^6^	1.83 × 10^−3^	2.48 × 10^−1^	1.00	1.00	1.00
4-methylcatechol	0.60	0.54	1.41 × 10^6^	1.44 × 10^6^	5.61 × 10^−1^	2.48 × 10^−1^	1.00	1.00	1.00
nicotiana-amine	0.60	0.55	3.44 × 10^7^	3.48 × 10^7^	9.67 × 10^−2^	2.54 × 10^−1^	1.00	1.00	0.99
1,3-propanediol	0.60	0.56	3.19 × 10^4^	8.27 × 10^4^	5.73 × 10^−1^	2.58 × 10^−1^	1.00	1.00	1.00
*N*-acetylserine	0.60	0.59	1.87 × 10^6^	1.07 × 10^6^	6.42 × 10^−2^	2.59 × 10^−1^	1.00	1.00	1.00
erythronate	0.60	0.64	9.27 × 10^4^	4.29 × 10^5^	4.38 × 10^−1^	2.60 × 10^−1^	1.00	1.00	1.00
1-palmitoyl-GPE	0.60	0.64	2.07 × 10^6^	3.41 × 10^6^	6.92 × 10^−1^	2.60 × 10^−1^	1.00	1.00	1.00
4-androsten-3beta,17beta-diol	0.60	0.61	3.81 × 10^6^	5.96 × 10^6^	6.02 × 10^−1^	2.60 × 10^−1^	1.00	1.00	1.00
